# The mediating role of psychological resilience in the relationship between physical exercise and sense of security among left-behind junior high school students: multi-group comparative analysis of only children and children with siblings

**DOI:** 10.3389/fpsyg.2024.1411175

**Published:** 2024-11-25

**Authors:** Qifei Xia, Qi Liu, Guoyou Qin

**Affiliations:** ^1^School of Physical Education, Ankang College, Ankang, China; ^2^Institute of Sports Training, Xi’an Institute of Physical Education, Xi’an, China; ^3^Qingshui County No. 5 Middle School, Tianshui, China; ^4^College of Physical Education, Hanjiang Normal University, Shiyan, China

**Keywords:** physical exercise, sense of security, psychological resilience, left-behind junior high school students, only children, children with siblings

## Abstract

**Background:**

This study aims to explore the mediating role of psychological resilience in the relationship between physical exercise and the sense of security among junior high school students, with a particular focus on variations based on only-child status.

**Methods:**

A survey was conducted among 649 left-behind junior high school students in Gansu Province, China, utilizing the Physical Activity Rating Scale-3 (PARS-3), Security Questionnaire (SQ), and Resilience Scale for Chinese Adolescents (RSCA).

**Results:**

Among the participants, the mean scores for physical exercise, psychological resilience, and sense of security were 40.78 ± 29.49, 51.14 ± 10.08, and 55.75 ± 14.35, respectively. A significant positive correlation was observed between physical exercise and sense of security (*r* = 0.210, *p* < 0.01), physical exercise and psychological resilience (*r* = 0.164, *p* < 0.01), and psychological resilience and sense of security (*r* = 0.443, *p* < 0.01). Mediation analysis revealed that psychological resilience partially mediates the effect of physical exercise on the sense of security, accounting for 33.9% of the total effect. Multi-group analysis indicated significant differences in this mediating effect between only children and children with siblings, with a stronger predictive role of psychological resilience for the sense of security among non-only children.

**Conclusion:**

Participation in physical exercise can promote the improvement of sense of security through the individual power factor of improving the psychological resilience of left-behind junior high school students, and this improvement effect is more significant for non-only children.

## 1 Introduction

The COVID-19 pandemic reduced daily travel activities, affecting general health and leading to a significant deficit in total physical activity. This decline contributed to negative psychological conditions, such as anxiety and stress, caused by feelings of insecurity and fear (Silva et al., 2020). Worldwide, public health physical activity guidelines emphasize children (usually 6–11 years old) and adolescents (12–19 years old) (Services and Branch, 1998). The international Convention on the Rights of the Child defines a child as any person under the age of 18 (UNCRC, 1989). Left-behind children refer to children whose parents (either one or both) have been away from their hometowns for work for at least 6 months without the provision of adequate family supervision (Zhao et al., 2014; Fellmeth et al., 2018). The latest official definition in China describes left-behind children as those under the age of 16 years old whose parents are absent due to work, or where one parent is absent, and the other is unable to provide proper guardianship, and who are unable to live normally with their parents (State Council of the PRC, 2016). It is estimated that by the turn of the millennium, about 24 million children under the age of 18 were left behind, a number expected to increase to 61 million in the following decade. This issue has raised concerns about their health and other social problems (Li et al., 2015). With the drastic increase in social pressure, the phenomenon of left-behind children has become a major social challenge in China (Lei et al., 2018).

A sense of security is defined as the anticipation of physical or mental danger, as well as the individual’s sense of powerlessness in coping with such risks, it mainly manifests as a sense of certainty and control (Zhong, 2004). A lower sense of psychological insecurity corresponds to a greater likelihood of emotional tension, timidity, vigilance, and a reluctance to engage socially (Liao et al., 2014). Humanistic theory (Hall and Lindzey, 1957) suggests that early adolescents are prone to a high degree of dependence on their parents due to personal incapacity, but as they age, the stability and durability of their sense of security changes with their objective environment, and the connection with their family decreases, making it necessary to create conditions that enhance self-security. In a “disturbed” life situation, adolescents often exhibit psychological characteristics such as sadness, shame, confusion, helplessness, etc. (Dursun et al., 2022). It has been found that the psychological security of left-behind children is significantly lower than that of non-left-behind children (Chen et al., 2020). These children habitually demonstrate fear, resistance and rejection in unfamiliar environments, with increasing uncertainty and a significant decrease in self-acceptance (Liu et al., 2023). In a sense, as young people come to become independent from their parents, the security issues of left-behind children are also becoming even more serious, and there is an urgent need for scientific and effective interventions to make up for this lack of security.

Previous research indicates that physical activity, as a routine social activity in which individuals form regular habits, can effectively promote a sense of security (Piestrzyński et al., 2021). For example, physical activities such as weight lifting, swimming, and walking trigger the release of endorphins, which produce relaxation and well-being (Abdulaziz Muhsen and Abdulaziz Muhsen, 2020), and also enhance memory, mood, and sleep, improving physical and mental health (Liddle et al., 2017; Al-Qahtani et al., 2018). For left-behind children, participation in various sports activities can give an interactive space where they can cooperate with and help each other, which fosters self-confidence, respect, care, and love (Shu and Wang, 2016). Another study has shown that exercise, as an important intervention, can alleviate adverse mood and behavioral disorders such as depression, anxiety and tension among adolescents (Rui et al., 2021). However, the global spread of COVID-19 has significantly reduced opportunities for adolescents to engage in physical activity (Salath et al., 2020). School curricula, including physical education, began to change from group-based to individual activities, primarily conducted online (Burke, 2020). Online exercise has become an important strategy, and one study points out that the exercise punch card model can be used to monitor and motivate students and help them maintain an interest in sports and avoid negative emotions such as fear, anxiety, and depression during the pandemic (Patriajati et al., 2020). A review of the existing literature reveals that no research has been conducted on the relationship between physical activity and the sense of security among Chinese left-behind students. Based on this gap, we hypothesize that physical exercise would help left-behind junior high school students control their self-defeating behaviors, regulate their emotional state, and overcome sociopathic barriers, thus increasing their sense of security. Accordingly, Hypothesis H1 is proposed:

H1: Physical activity positively predicts a sense of security among left-behind junior high school students.

Resilience refers to an individual’s ability to self-regulate or pressures to flexibly adapt to complex and changing environments when confronted with different pressures or challenges (Mak et al., 2011). The mental resilience process model suggests that resilience develops a combination of increasing protective factors and external risk factors acting simultaneously after a stressful situational event (Richardson, 2002). Two main areas include individual human factors (goal focus, emotional control, positive cognition) and supportive factors (family support, interpersonal assistance) (Hu and Gan, 2008). Among them, personal human factors act as a positive source of motivation and contribute to the positive development of the individual in the face of adversity (Fengjun et al., 2022). These factors are key to individual resistance to external disturbances and the level of self-confidence. According to exercise psychology, physical activity has a positive effect on attentional stability (De Greeff et al., 2018), negative emotion regulation (VanKim and Nelson, 2013), and cognitive abilities (Donnelly et al., 2016). From a constructivist perspective, resilience theory suggests that the development of resilience is closely related to an individual’s mental health, physical functioning, and social adaptability (Yang, 2014). Existing research supports the idea that participation in physical activity enhances physical fitness and serves as a protective factor for increased mental toughness (Guo and Liang, 2023). Psychological resilience acts as a buffer against stress (Liang, 2019), and when faced with setbacks, this internal drive may generate positive feedback that promotes psychological fulfillment and a sense of belonging. Previous research has found that individuals with higher levels of psychological resilience have a greater sense of control over their lives and a greater belief in their ability to manage adversity, which in turn enhances their sense of security (Yang et al., 2021). Accordingly, the following hypotheses are proposed:

H2a: Physical activity positively predicts the resilience of left-behind junior high school students; H2b: Resilience positively predicts the sense of security of left-behind junior high school students.

In addition, there is a need to further explore the variability in physical activity, mental toughness, and sense of security with respect to lone-child status. Research suggests that only children who do not have to share or compete for parental attention and resources like other families with siblings benefit from the emotional warmth and support provided by parents through positive parenting (Morgan et al., 2020). Studies have found that children with siblings have higher levels of moderate to vigorous physical activity (MVPA) (Kracht and Sisson, 2018). As adolescents grow older, their participation in sports increases with the presence of siblings and other important family members (Santos et al., 2023). Furthermore, research shows that only children score higher in security measures than non-only children (Liao et al., 2014). According to attachment theory, only children, who often receive attention and protection from an early age, may develop a greater sense of security due to their parents’ increased responsiveness and attention and responsiveness (Liu et al., 2010). A comparison of the areas where the one-child policy was implemented with those where the non-one-child policy was implemented revealed that non-one-child children had significantly higher levels of fear, anxiety and depression than one-child children (Yang et al., 1995). Accordingly, the following research hypothesis H3 is proposed:

H3: Under the influence of sole-child status, there is a difference in the mediating effect of resilience between physical activity and left-behind junior high school students’ sense of security.

In summary, the sense of security among left-behind children has become a critical social concern, especially in remote areas of China. Exploring the relationship between physical activity and a sense of security, as well as the mediating role of individual human factors, can provide a basis for promoting the healthy growth of left-behind children.

## 2 Methods

### 2.1 Participants and procedures

China’s provinces and regions are characterized by dichotomy, and Gansu Province is a relatively backward region in the western part of the country in terms of the level of economic development, with a large number of left-behind children, children from single-parent families, and other special groups in general (Robinson, 2016). Utilizing a convenience sampling method, this study targeted left-behind junior high school students from five schools in Gansu Province, China, all under 18 years of age. The second author led the survey process, which began with obtaining written informed consent from the participants and their class teachers. To ensure confidentiality and anonymity, students signed agreements in the classroom before the questionnaire distribution and collection, with all procedures designed to protect participant privacy. Out of 700 distributed questionnaires, 649 were deemed valid, yielding a response rate of 92.7%, the mean age was 12.82 ± 1.23. The demographic breakdown included 292 males (45%) and 357 females (55%), with 165 students in the first year (25.4%), 337 in the second year (51.9%), and 147 in the third year (22.7%). Urban residents accounted for 392 students (60.4%), while rural residents made up 257 students (39.6%). Additionally, the sample comprised 330 children (50.8%) and 319 children with siblings (49.2%).

### 2.2 Measures

#### 2.2.1 Physical activity rating scale

The physical exercise scale, originally developed by the Japanese scholar Kimitaka Hashimoto and subsequently revised by Chinese researcher Deqing Liang et al., assesses the overall level of physical activity (Liang, 1994). This instrument measures the intensity, frequency, and duration of physical exercise, calculating the total exercise amount as the product of these three factors. Intensity and frequency are each scored on a scale from 1 to 5, categorized into five levels, while duration is scored from 0 to 4. The exercise volume is classified as low (≤ 19 points), moderate (20–42 points), or high (≥43 points). The reliability of this scale was confirmed in the current study, with a Cronbach’s *α* coefficient of 0.775.

#### 2.2.2 Security questionnaire

The Sense of Security Scale, developed by Zhong Cong of the Beijing Institute of Mental Health and An Lijuan of Hebei Normal University, was utilized in this study (Zhong, 2004). This instrument comprises 16 items distributed across two dimensions: interpersonal sense of security and certainty of control, with the overall sense of security being the aggregate of these two dimensions. Responses were measured using a five-point Likert scale, ranging from “strongly agree” to “strongly disagree,” where higher scores indicate a greater level of sense of security. The scale’s reliability was confirmed in this study, evidenced by a Cronbach’s alpha coefficient of 0.954, with internal consistency coefficients of 0.911 and 0.914 for the respective dimensions. Confirmatory factor analysis yielded satisfactory fit indices: *x*^2^/df = 3.203, GFI = 0.931, RMSEA = 0.058, CFI = 0.967, and SRMR = 0.028.

#### 2.2.3 Psychological resilience scale for adolescents

The study employed a psychological resilience scale developed by Hu and Gan (2008), consisting of 27 items across five dimensions. The initial three dimensions assess aspects of individual power, including goal focus, emotional control, and positive cognition, while the remaining two dimensions evaluate support factors, namely family support and interpersonal assistance. Given the scale’s bifurcation into personal qualities and social influences, this research focused exclusively on the first three factors pertinent to individual power. A five-point Likert scale was used for responses, indicating that higher scores reflect greater levels of Psychological resilience. The internal consistency of the scale was robust in this study, with a Cronbach’s alpha coefficient of 0.909 for the overall scale, and the coefficients for the individual power and support strength dimensions were 0.857 and 0.815, respectively.

### 2.3 Analytical strategy

Data analysis was conducted using SPSS version 23.0, beginning with Harman’s single-factor test to address potential common method bias, followed by descriptive statistics, independent samples t-tests, one-way ANOVA, and correlation analyses, with *p* < 0.05 indicating statistical significance. The PROCESS macro developed by Hayes was employed for statistical analysis, including mediation effect testing with bias-corrected bootstrap confidence intervals (5,000 resamples). Furthermore, AMOS version 26.0 was utilized to examine the mediation effect of psychological resilience (individual power) in the relationship between physical exercise and sense of security, along with conducting multi-group comparative analyses.

## 3 Results

### 3.1 Common method bias test

To assess the potential for common method bias, inherent in data derived from subjective questionnaire surveys, Harman’s single-factor test was applied (Zhou and Long, 2004). This involved conducting an exploratory factor analysis on all items from the scales measuring physical exercise, sense of security, and mental resilience without rotation. The analysis identified six components with eigenvalues greater than 1, where the largest component accounted for 33.68% of the variance, falling below the critical threshold of 40%. This suggests that common method bias does not significantly affect the study’s findings.

### 3.2 Tests for variability in different demographic characteristics of participants

Independent samples *t*-tests were conducted to examine gender and place of origin differences in physical exercise, mental resilience, and sense of security among left-behind junior high school students. The results, as outlined in Table 1, indicated no significant gender differences across the measures of physical exercise, mental resilience, or any dimensions of a sense of security. However, significant differences based on the place of origin were observed for psychological resilience (individual power) and the goal focus dimension (*p* < 0.05), with urban students scoring significantly higher than their rural counterparts. Further analysis using one-way ANOVA on the variable of grade level revealed significant differences in the sense of security and the dimension of certainty control across grades (*p* < 0.05), as detailed in Table 2. Specifically, students in the second year of junior high school exhibited significantly higher scores compared to those in the first and third years.

**Table 1:** Difference test for each variable on gender, birthplace.

Variables	Gender		*p*	Birthplace		*p*
	Boys	Girls		Rural	Urban	
Physical exercise	41.91 ± 30.75	39.86 ± 28.43	0.377	40.03 ± 29.58	41.93 ± 29.37	0.421
Sense of security	56.43 ± 14.79	55.20 ± 13.98	0.277	54.93 ± 14.55	57.01 ± 13.99	0.071
Sense of certainty	28.30 ± 7.53	27.65 ± 7.07	0.257	27.55 ± 7.31	28.54 ± 7.21	0.088
Sense of control	28. 13 ± 7.59	27.55 ± 7.24	0.320	27.38 ± 7.58	28.46 ± 7.08	0.067
Individual power	50.76 ± 9.83	51.46 ± 10.30	0.378	50.51 ± 10.18	52. 11 ± 9.88	0.048^*^
Goal focus	17.45 ± 3.89	17.70 ± 3.99	0.419	17.28 ± 3.98	18.05 ± 3.84	0.015^*^
Emotional control	19.55 ± 5.03	20.05 ± 5. 12	0.211	19.60 ± 4.95	20. 16 ± 5.27	0.174
Active cognition	13.76 ± 3.38	13.71 ± 3.26	0.842	13.63 ± 3.38	13.90 ± 3.21	0.297

**Table 2:** Tests of difference in variables at grade levels.

Variables	Grade			*p*
	Grade 1	Grade 2	Grade 3	
Physical exercise	44.08 ± 29.52	38.95 ± 30.16	41.28 ± 27.73	0.183
Sense of security	56.07 ± 15.03	56.76 ± 14.88	53.08 ± 11.90	0.033^*^
sense of certainty	28. 12 ± 7.67	28.39 ± 7.53	26.71 ± 6.07	0.062
sense of control	27.95 ± 7.64	28.37 ± 7.68	26.37 ± 6.23	0.023^*^
Individual power	50.35 ± 11.39	51.25 ± 10.57	51.82 ± 6.89	0.423
Goal focus	17.08 ± 4.18	17.65 ± 4. 11	18.01 ± 3.15	0.103
Emotional control	19.95 ± 5.82	19.79 ± 5.26	19.77 ± 3.61	0.933
Active cognition	13.32 ± 3.55	13.81 ± 3.49	14.03 ± 2.50	0.134

### 3.3 Correlation analysis

Pearson correlation analysis was employed to explore the relationships among physical exercise, psychological resilience (individual power), and sense of security, along with their respective dimensions. The results, detailed in Table 3, demonstrated significant positive correlations across all examined variables. Specifically, physical exercise showed a significant positive correlation with both the overall sense of security and its dimensions (*p* < 0.01), as well as with psychological resilience (individual power) (*p* < 0.01). Furthermore, psychological resilience (individual power) was significantly positively correlated with the overall sense of security and its dimensions (*p* < 0.01).

**Table 3:** Correlation test between variables.

Variables	1	2	3	4	5	6	7	8
Physical exercise	1.000							
Sense of security	0.210^**^	1.000						
sense of certainty	0.212^**^	0.978^**^	1.000					
sense of control	0.198^**^	0.978^**^	0.913^**^	1.000				
Individual power	0.164^**^	0.443^**^	0.425^**^	0.442^**^	1.000			
Goal focus	0.117^**^	0.497^**^	0.483^**^	0.489^**^	0.863^**^	1.000		
Emotional control	0.176^**^	0.256^**^	0.241^**^	0.260^**^	0.807^**^	0.480^**^	1.000	
Active cognition	0.088^*^	0.364^**^	0.348^**^	0.364^**^	0.779^**^	0.701^**^	0.351^**^	1.000

### 3.4 Partial mediating role of psychological resilience

According to the mediation effect testing method (Wen et al., 2004), the PROCESS macro (Model 4) in SPSS was used to assess the mediating role of psychological resilience (individual power) in the relationship between physical exercise and sense of security among left-behind junior high school students. Controlling for gender, place of origin, and grade level, physical exercise was initially tested as a predictor of a sense of security, revealing a significant positive effect (*β* = 0.206, *p* < 0.01). Subsequently, a regression analysis predicted psychological resilience (individual power) from physical exercise, showing a significant positive relationship (*β* = 0.165, *p* < 0.01). In the final step, with both physical exercise and psychological resilience (individual power) as predictors, the analysis confirmed their significant positive predictive effects on a sense of security (*β* = 0.136, *p* < 0.01 for physical exercise; *β* = 0.424, *p* < 0.01 for psychological resilience), as detailed in Table 4.

**Table 4:** Mediation model tests for physical activity, psychological resilience (individual power), and perceived safety.

Outcome variable	Predictor variable	*R*	*R^2^*	*F*	*B*	*β*	*t*
Sense of security	Physical exercise	0.057	0.049	7.717	0.100	0.206	5.357^**^
Individual power	Physical exercise	0.036	0.028	4.780	0.056	0.165	4.239^**^
Sense of security	Individual power	0.230	0.223	31.924	0.603	0.424	12.015^**^
	Physical exercise				0.066	0.136	3.862^**^

The mediation effect of psychological resilience (individual power) in the relationship between physical exercise and a sense of security among left-behind junior high school students was evaluated using a bias-corrected bootstrap method with 5,000 samples, setting a 95% confidence interval. Results, as shown in Table 5, indicated that the indirect path from physical exercise through psychological resilience (individual power) to a sense of security was significant, with an estimated indirect effect of 0.034 and a 95% confidence interval [CI: 0.031–0.110], which does not include zero. The mediation effect accounted for 33.9% of the total effect, suggesting that psychological resilience (individual power) plays a significant partial mediating role between physical exercise and a sense of security.

**Table 5:** Results of the mediation effect test for psychological resilience (individual power).

681 effect	Effect value	Boot standard error	Boot 95% CI lower	Boot 95% CI upper	Percentage
Total effect	0.100	0.019	0.064	0.137	
Direct effect	0.066	0.013	0.033	0.100	66. 1%
Indirect effect	0.034	0.020	0.031	0.110	33.9%

**Figure 1:**
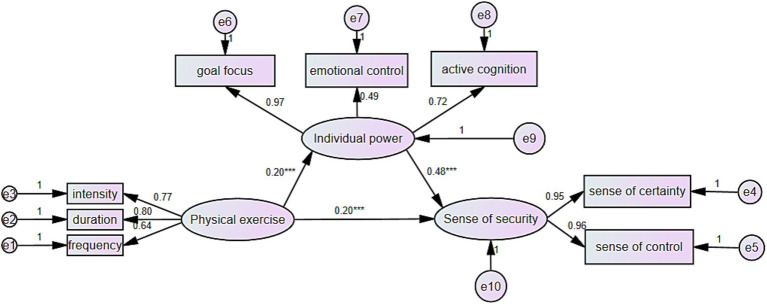
The mediating effect of psychological resilience between physical exercise and left-behind middle school students’ sense of security. ^***^p < 0.001, ^**^p < 0.01, ^*^p < 0.05.

To further substantiate the mediating role of psychological resilience (individual power) between physical exercise and a sense of security among left-behind students, a fit analysis of the hypothesized model was conducted using AMOS version 26.0. The model demonstrated excellent fit, with the following indices: degrees of freedom (DF) = 17, chi-square to degrees of freedom ratio (x^2^/df) = 1.518, Comparative Fit Index (CFI) = 0.997, Normed Fit Index (NFI) = 0.990, Relative Fit Index (RFI) = 0.997, Incremental Fit Index (IFI) = 0.997, and Root Mean Square Error of Approximation (RMSEA) = 0.028, all indicating acceptable fit levels. Specifically, physical exercise significantly predicted psychological resilience (individual power) (*β* = 0.199, *p* < 0.001) and a sense of security (*β* = 0.202, *p* < 0.001). Furthermore, psychological resilience (individual power) significantly predicted a sense of security (*β* = 0.483, *p* < 0.001), with a 95% confidence interval [CI: 0.043–0.160] excluding zero. These results validate the mediation effect of psychological resilience (individual power) in the relationship between physical exercise and a sense of security (see Figure 1).

### 3.5 Multi-cluster test analysis

**Figure 2:**
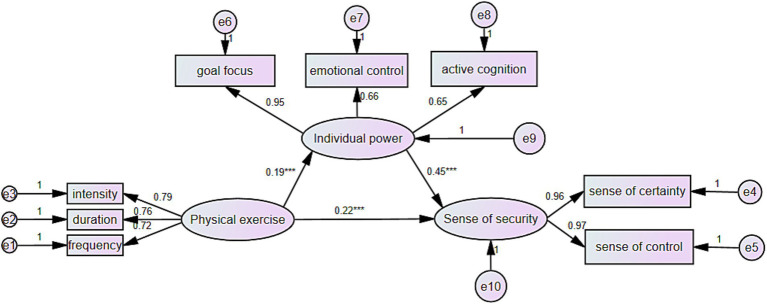
Mediating effect of psychological resilience between physical exercise and sense of security of left-behind junior high school students (lone students). ^***^p < 0.001, ^**^p < 0.01, ^*^p < 0.05.

**Figure 3:**
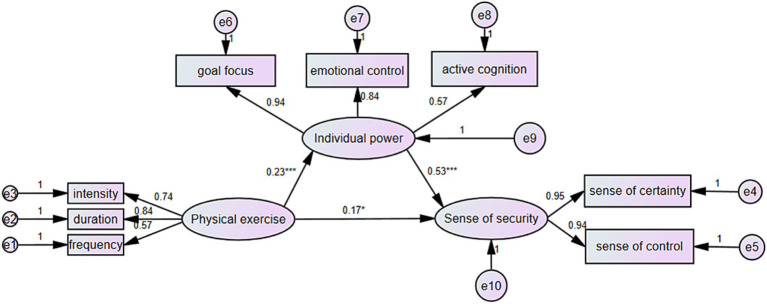
The mediating effect of psychological resilience on the relationship between physical activity and left-behind junior high school students’ sense of security (non-alone students). ^***^p < 0.001, ^**^p < 0.01, ^*^p < 0.05.

This study also explored the differential mediating role of psychological resilience (individual power) between physical exercise and a sense of security among left-behind junior high school students based on their only-child status. Initial model fit indices for only children and children with siblings indicated both models were suitable for further multi-group comparative analysis. The fit indices were favorable: for only children, DF = 17, *x*^2^/df = 2.561, CFI = 0.982, NFI = 0.971, RFI = 0.953, IFI = 0.982, RMSEA = 0.069; for children with siblings, DF = 17, *x*^2^/df = 1.552, CFI = 0.991, NFI = 0.976, RFI = 0.961, IFI = 0.991, RMSEA = 0.042 (see Figures 2, 3). Subsequent multi-group analysis using structural equation modeling to compare models across groups revealed no significant differences in model fit for most comparisons (Δ*x*^2^/df between models M1 and M2, M3 and M4, and M5 and M6, all *p* > 0.05). However, significant differences emerged between models M2 and M3 (Δ*x*^2^/df = 2.03, *p* < 0.05) and between M4 and M5 (Δ*x*^2^/df = 9.461, *p* < 0.001), with changes in TLI and CFI indicating significant structural weight and residual model differences across only-child status groups. Further analysis showed that while physical exercise significantly predicted psychological resilience (individual power) in both groups, the path coefficient’s difference was not significant (CR = 0.685, *p* > 0.05). Similarly, no significant difference was found in the impact of physical exercise on a sense of security across only-child status (CR = −1.845, *p* > 0.05). However, the path from psychological resilience (individual power) to a sense of security showed significant variation (CR = 2.518, *p* < 0.05), with children with siblings demonstrating a stronger relationship (*β* = 0.533, *p* < 0.001) compared to only children (*β* = 0.453, *p* < 0.001), indicating a stronger predictive power of psychological resilience on a sense of security among children with siblings (Table 6).

**Table 6:** Adaptation table for multi-cluster analysis.

Model	*x* ^2^	DF	*x*^2^/DF	NFI	IFI	TCL	CFI	RMSEA	Δ(*x*^2^Δdf)
M1	68.246	32	2.133	0.974	0.986	0.975	0.986	0.042	
M2	77.761	37	2.102	0.971	0.984	0.976	0.984	0.041	9.515(5)
M3	100.100	48	2.085	0.962	0.980	0.977	0.980	0.041	22.339(11)
M4	100.257	49	2.046	0.962	0.980	0.977	0.980	0.040	0. 158(1)
M5	119.179	51	2.337	0.955	0.974	0.971	0.974	0.045	18.922(2)
M6	134.363	59	2.277	0.949	0.971	0.972	0.971	0.044	15. 184(8)

M1 is the unrestricted model, M2 is the measurement weight model, M3 is the structural weight model, M4 is the structural covariance model, M5 is the structural residual model, and M6 is the measurement residual model.

## 4 Discussion

### 4.1 Differences in different demographic characteristics of middle school students left behind

The present study found no gender differences in physical activity among left-behind junior high school students, but boys’ scores were higher than girls’ scores, which is basically the same as the results of previous studies (Fu and Zhao, 2020). This may be due to the fact that boys are more inclined to experience exciting and challenging sports, while girls often show a gentle, quiet and stable character and are willing to participate in less intense, relaxing and enjoyable social sports activities (Lever, 2020). Furthermore, the study identifies significant disparities in mental resilience and goal-focused attention among these students based on their geographical backgrounds, with urban students outperforming their rural counterparts. According to Ecological Systems Theory (Bronfenbrenner and Evans, 2000), individuals are embedded within various environmental systems that foster positive interactions, influencing the acquisition of knowledge and experience. The psychological environment and resource conditions of urban left-behind students are significantly better than those of rural left-behind students, they can obtain more education and family material resources, make full use of the existing conditions to formulate a clear study plan, take their learning attitude seriously, and concentrate more on their concentration, while rural left-behind junior high school students have a difficult life since childhood and lack corresponding protective resources, especially after entering the junior high school stage, the comparison of classmates and friends becomes more and more serious, and problems such as poor learning interest and deviation in learning goal positioning will occur (Sun et al., 2015).

The study also notes significant differences in the sense of security and perceived control among different grades. Second-year students, having spent more time in school, exhibit higher independence, and adaptability to the school’s environment, and have established positive interpersonal relationships, showcasing a preference for self-expression. First-year students, still in early adolescence with lower self-awareness, struggle with new and unfamiliar environments. Third-year students score the lowest, likely due to the increased academic pressure of preparing for entrance exams, leading to frequent encounters with the uncertainties of present and future academic performance and stability (Du et al., 2019).

### 4.2 The relationship between physical exercise, psychological resilience, and sense of security among left-behind middle school students

This study reveals a significant positive correlation between physical exercise and the sense of security among left-behind middle school students, corroborating previous findings (Piestrzyński et al., 2021). Psychoanalyst Horney (2013) suggested that familial discord leads to psychological anxiety, contributing to a diminished sense of security, a notion paralleled in Freud’s psychoanalytic theory (Freud, 1936), which identifies insecurity as an intrinsic motivator for anxiety symptoms. Anxiety, a key predictor of security levels, often prompts avoidance behaviors or perceived rejection risks in socially anxious individuals (Calfas and Taylor, 1994). Physical exercise has been shown to mitigate anxiety, enhance self-esteem, and foster positive cognition in left-behind middle school students, thereby improving psychological well-being (Ren and Li, 2020). Humanistic psychologist Erich Fromm argued that the process of growing up involves detachment from family bonds, increasing feelings of loneliness and helplessness, thereby exacerbating security deficits (Ryckman, 1989). Studies indicate that engagement in physical activities can enhance social and interpersonal relationships among adolescents, potentially preventing or reducing loneliness and fostering a sense of control and security (Pinto et al., 2021). For left-behind middle school students undergoing puberty, physical exercise can bolster social skills, counteract isolation, and enhance their sense of security (Bernardon et al., 2011). Moreover, feelings of interpersonal alienation and loneliness in social contexts are significant factors affecting these students’ sense of security. This research enriches the body of knowledge on the impact of physical exercise on the sense of security, offering theoretical and practical insights for enhancing the well-being of left-behind middle school students through physical activities.

Research further indicates a significant positive correlation between physical exercise and psychological resilience, suggesting that active engagement in physical exercise enhances concentration, emotional control, and cognitive capabilities, aligning with findings by Dunston et al. (2022). Engaging in sports activities of varied intensity, frequency, and duration during adolescence, such as aerobic fitness, can elevate resilience by fostering the neuroregulatory mechanisms of self-regulation, thereby mitigating the risk of psychological health issues (Belcher et al., 2021). This corroborates the perspective of physical exercise as a beneficial intervention, where higher levels of individual exercise correlate with increased capacity to manage setbacks, optimistically navigate life’s challenges, and transform adverse conditions into favorable outcomes, thus cultivating positive personal qualities. The significant positive relationship between psychological resilience and a sense of security indicates that higher levels of “individual power” within psychological resilience are associated with enhanced feelings of security, highlighting psychological resilience as a critical factor influencing interpersonal security and a sense of control. Left-behind middle school students, often raised by grandparents or other guardians, develop independence and self-reliance from an early age, predisposing them to analytical problem-solving (Xu et al., 2013). Research reveals that children with higher levels of psychological resilience (Glantz and Johnson, 1999) possess superior emotional regulation and positive cognition, tending not to blame themselves for their circumstances but rather actively seeking solutions, thereby experiencing a greater sense of freedom and control over their lives. The Kumpfer psychological resilience theoretical model suggests that resilience levels rise upon facing and overcoming significant stressors and challenges, aiding individuals in using protective factors to regulate psychological stress and positively predict outcomes under high-risk conditions (Shi et al., 2016). Rew et al. found that individuals with higher mental resilience are more likely to rely on themselves, considering resilience as an adaptive strategy or a defense against loneliness and despair (Rew et al., 2001). It is noteworthy that some left-behind middle school students may experience psychological trauma in childhood, lacking adequate self-protection. Rutter identified mental resilience as a crucial protective factor in the effective treatment and prevention of psychiatric disorders, indicating that individuals with high levels of mental resilience can effectively alleviate and improve adverse psychological symptoms (Rutter, 1985). Therefore, it is imperative to focus on nurturing the personal qualities of left-behind middle school students, leveraging their inherent strengths and talents to proactively manage stress and crises, thereby maintaining optimal mental health and enhancing their sense of security.

### 4.3 The mediating role of psychological resilience in the relationship between physical exercise and feelings of safety in remaining middle school students

Mediation analysis reveals that psychological resilience (individual power) plays a partial mediating role between physical exercise and the sense of security among left-behind middle school students. This finding confirms Hypothesis H2, illustrating that physical exercise influences these students’ sense of security through enhancing levels of psychological resilience (encompassing goal focus, emotional control, and positive cognition). Garmezy (1993) categorizes psychological resilience models into compensatory, challenge, and vulnerability models. Particularly, the challenge model suggests that when the difficulty of life’s tasks is moderate, individuals often judge based on their capabilities, thereby enhancing self-efficacy in addressing and resolving uncompleted tasks, potentially benefiting psychological health growth. Setting appropriate exercise goals can ignite intrinsic motivation for physical activity, enabling individuals to overcome multiple barriers, improve physical capabilities, and develop resilience and determination, thus achieving the benefits of physical fitness. From the perspective of positive psychology in sports, physical exercise offers a platform for self-expression, cultivating positive psychological constructs such as psychological resilience, perseverance, and flexibility to mitigate the negative effects of emotional dysregulation (Mann and Narula, 2017). Consequently, society should intensify psychological support for left-behind students, fostering resilience and perseverance through diverse sports activities, encouraging positive self-assessment, and nurturing an optimistic and confident outlook toward complex social changes and the future.

### 4.4 Multi-group comparative analysis of the mediating effect of psychological resilience

The multi-group analysis indicates variability in the mediation model based on the only-child status, with psychological resilience (individual power) exerting a stronger predictive effect on the sense of security among children with siblings (Xiao et al., 2019). This variation likely stems from differences in family environments and parental upbringing styles. Research has shown that sibling relationships among left-behind children positively correlate with psychological resilience. The interaction among non-only children fosters mutual understanding of emotions, thoughts, and intentions, and experiences of competition and cooperation from a young age enhance resilience through adversity (McHale et al., 2012). In contrast, only children, lacking sibling interactions, tend to be more self-centered in problem-solving and exhibit lower levels of social support, often relying excessively on parents or relatives for problem analysis and resolution (Gutgesell and Payne, 2004). For middle school-aged left-behind students, adolescence entails the maturation of body image, abstract thinking, and cognitive functions. However, due to parents working away, the parent–child relationship fails to improve and maintain adequately, leading to deficiencies in coping abilities and social skills, which may indirectly cause a psychological sense of security imbalance. Non-only children, on the other hand, can compensate for this with sibling relationships. Psychological resilience encompasses three factors: individual, environmental, and community aspects. From an individual trait perspective, facing significant stress or danger with good adaptability allows for setting firmer goals and calmly tackling academic tasks in future learning, satisfying the need for a sense of security. Compared to only children, left-behind students with siblings are better at pooling ideas, mutually honing each other, setting reasonable learning plans, having a clear understanding of difficulties, potentially experiencing less helplessness or emotional dysregulation, and valuing competition and sharing for experience exchange to enhance their sense of security. Family size and educational approaches lead to distinctive traits in only children, such as dominance, anxiety, quarrelsomeness, or overprotectiveness, exhibiting emotional instability during conflicts (Blake, 1981). Many left-behind only children may live in single-parent families or under grandparent care for extended periods, bearing more household or caregiving responsibilities than their non-left-behind peers, often accumulating disappointment and dissatisfaction with their families. Consequently, they might exhibit resistance and rejection in unfamiliar environments or social interactions, requiring a longer period to adapt in terms of emotional control and positive cognition.

This study also acknowledges certain limitations and areas for improvement: (1) It is based on a cross-sectional survey conducted among junior high school students left behind during the pandemic, primarily using self-reported data, which has its constraints. Future research should include longitudinal studies to address these limitations. (2) The survey’s geographic scope was restricted to specific regions, potentially limited by time and space, and the sample size was insufficient. Future empirical studies should consider a comprehensive comparison of data across different regions and stages, including urban students left behind, to enhance the persuasiveness of the findings. (3) The use of a physical exercise scale for measuring may not allow for regular tracking of the students’ exercise levels. Future research should incorporate periodic physical intervention experiments to make the research design more scientific and standardized.

## 5 Conclusion

Our study demonstrates that physical exercise significantly enhances the sense of security among junior high students in Gansu Province, offering an effective intervention strategy for bolstering the psychological well-being of children in China’s impoverished regions. Furthermore, we identified that individual power, a component of psychological resilience, mediates the relationship between physical exercise and the student’s sense of security. Diverse exercise activities notably improve focus, emotional control, and positive cognition, serving as a critical link in enhancing the sense of security. Notably, this mediating effect varies with sibling status, where the predictive power of individual power on sense of security is stronger among students with siblings. These findings underscore the necessity for further exploration into scientifically based adolescent exercise programs to optimize mental health benefits.

## Data Availability

The original contributions presented in the study are included in the article/supplementary material, further inquiries can be directed to the corresponding author/s.
